# Septal stimulation attenuates hippocampal seizure with subregion specificity

**DOI:** 10.1002/epi4.12983

**Published:** 2024-06-03

**Authors:** Qingyang Zhang, Yu Wang, Fei Wang, Dongxiao Jiang, Yingjie Song, Lin Yang, Mengdi Zhang, Yi Wang, Yeping Ruan, Jiajia Fang, Fan Fei

**Affiliations:** ^1^ Key Laboratory of Neuropharmacology and Translational Medicine of Zhejiang Province, Department of Neurology, The First Affiliated Hospital, School of Pharmaceutical Sciences Zhejiang Chinese Medical University Hangzhou China; ^2^ Chinese Medicine Plant Essential Oil Zhejiang Engineering Research Center, School of Pharmaceutical Sciences Zhejiang Chinese Medical University Hangzhou China; ^3^ Department of Neurology, The Fourth Affiliated Hospital, School of Medicine Zhejiang University Yiwu China

**Keywords:** cholinergic neuron, deep brain stimulation, hippocampal rhythm, seizure, septum

## Abstract

**Objective:**

Deep brain stimulation (DBS) is a promising approach for the treatment of epilepsy. However, the optimal target for DBS and underlying mechanisms are still not clear. Here, we compared the therapeutic effects of DBS on distinct septal subregions, aimed to find the precise targets of septal DBS and related mechanisms for the clinical treatment.

**Methods:**

Assisted by behavioral test, electroencephalography (EEG) recording and analyzing, selectively neuronal manipulation and immunohistochemistry, we assessed the effects of DBS on the three septal subregions in kainic acid (KA)‐induced mouse seizure model.

**Results:**

DBS in the medial septum (MS) not only delayed generalized seizure (GS) development, but reduced the severity; DBS in the vertical diagonal band of Broca (VDB) only reduced the severity of GS, while DBS in the horizontal diagonal band of Broca (HDB) subregion showed no anti‐seizure effect. Notably, DBS in the MS much more efficiently decreased abnormal activation of hippocampal neurons. EEG spectrum analysis indicated that DBS in the MS and VDB subregions mainly increased the basal hippocampal low‐frequency (delta and theta) rhythm. Furthermore, ablation of cholinergic neurons in the MS and VDB subregions blocked the anti‐seizure and EEG‐modulating effects of septal DBS, suggesting the seizure‐alleviating effect of DBS was dependent on local cholinergic neurons.

**Significance:**

DBS in the MS and VDB, rather than HDB, attenuates hippocampal seizure by activation of cholinergic neurons‐augmented hippocampal delta/theta rhythm. This may be of great therapeutic significance for the clinical treatment of epilepsy with septal DBS.

**Plain Language Summary:**

The optical target of deep brain stimulation in the septum is still not clear. This study demonstrated that stimulation in the medial septum and vertical diagonal band of Broca subregions, but not the horizontal diagonal band of Broca, could alleviate hippocampal seizure through cholinergic neurons‐augmented hippocampal delta/theta rhythm. This study may shed light on the importance of precise regulation of deep brain stimulation therapy in treating epileptic seizures.

**Key points**

- Deep brain stimulation (DBS) in the MS and VDB, rather than the HDB, attenuates hippocampal seizure.
- DBS in the MS efficiently decreases aberrant neuronal activation of hippocampal neurons.
- Anti‐seizure effects of DBS in the MS and VDB depend on local cholinergic neuron‐mediated hippocampal low‐frequency rhythm.

## 1 INTRODUCTION

Temporal lobe epilepsy (TLE) is a common type of refractory epilepsy,^1^, ^2^ with the drug resistance rate exceeding 70%.^3^ Although a large proportion of patients with refractory TLE could be well controlled by neurosurgery,^4^, ^5^ it is not suitable in all cases and sometimes even induces irreversible neural damages. For those patients, deep brain stimulation (DBS) is a promising alternative approach.^6^ There are a variety of potential targets in the brain for DBS treatment, including the anterior nucleus of the thalamus (ANT), hippocampus, centromedian nucleus of the thalamus (CMT), subthalamic nuclei, and others.^7^, ^8^, ^9^ Among them, the ANT has become the most widely used target, reducing the incidence of seizures by 54%.^10^ However, not all patients underwent ANT‐DBS received favorable results and some even became worse.^11^ Psychiatric adverse effects of ANT‐DBS, which may be due to the tight connection between the ANT and the anterior cingulate cortex or orbitomedial prefrontal cortex, were also reported.^12^ Additionally, other targets like hippocampus and CMT were also reported to reduce the frequency of seizures by 30%–90% and 57%, respectively,^13^, ^14^ whereas therapeutic efficacy of DBS in these two targets shows individual variance and they also disrupt normal functions.^9^ Thus, there is still plenty of possibility to improve therapeutic efficacy and eschew side effects of DBS treatment by finding more optimized targets and exploring relevant mechanisms.

The septum is located in the basal, posterior frontal lobe in the midline, consisting of cholinergic, glutamatergic, and GABAergic neurons.^15^ It extensively projects to multiple cortical and subcortical regions, like the hippocampus and the parahippocampal cortex, which are closely associated with TLE.^16^, ^17^ Therefore, the septum has been evaluated in animal studies as an alternative DBS target for the treatment of TLE. For instance, DBS in the septum increases the seizure threshold and improves cognition in the pilocarpine‐induced seizure model.^18^ Septal DBS also terminates epileptic seizures and prevents seizure susceptibility in rat kindling model.^19^ Our previous study also showed that DBS targeting septum was highly effective with wide range of stimulation frequency in KA‐induced acute seizure model and hippocampal‐kindled epilepsy model through entrainment of the hippocampal theta rhythm.^20^ Interestingly, although most studies subscribed the anti‐seizure effect of septal DBS, our previous study showed the effect could vary significantly among individual animals. This reminded us precise subregion should be taken into consideration when analyzing effects of septal DBS.

Anatomically, the septum is a large brain region, comprising the medial septum (MS) and vertical and horizontal diagonal band of Broca (VDB and HDB).^21^ These three subregions consist of biased distribution of different neuronal types. In rodents, about 42% of cholinergic neurons in the septum are uniformly concentrated in the MS and others are GABAergic and glutamatergic neurons, which are present in the medial and lateral parts of the MS, respectively.^22^, ^23^ The rest of 58% of cholinergic neurons are distributed in the VDB and HDB, mainly lateral and caudal to the limb of the HDB, while GABAergic and glutamatergic neurons are slightly higher in the VDB and much lower in the HDB.^24^, ^25^ Such biased distribution suggests there may exist difference in regulation of physiological and pathological functions of these three septal subregions. Indeed, they may play distinct roles in epilepsy. Although early neuronal death in the septum was reported,^26^ continuous stomata swelling and conspicuous neuronal damage were only observed in the MS in pilocarpine‐induced seizures,^27^ rather than the VDB and HDB. In addition, MRI research has demonstrated that patients with TLE have a reduced volume of the MS,^28^ and furthermore, the study found that direct MS‐hippocampus cholinergic circuit alleviates TLE by driving the downstream somatostatin effector.^29^ Moreover, hippocampal theta oscillation induced by chemical or electrical stimulation of the MS alleviates seizures induced by pentylenetetrazol.^30^ The activation of PV interneurons in the MS abates pilocarpine‐induced seizures, further suggesting that the MS also plays a role in the occurrence of focal epilepsy.^31^ These findings collectively support that the changes in neuroplasticity in the MS contribute to epileptogenesis. Compared with the MS, how the VDB and HDB function in epilepsy remains largely exclusive.

In the present study, we compared the anti‐seizure effects of DBS in distinct subregions of the septum and the underlying mechanisms. Our results suggested that DBS in the MS and VDB alleviates KA‐induced seizure, while HDB‐DBS does not. Furthermore, the effect of DBS in the MS and VDB relies on cholinergic neurons and thereby modulates hippocampal rhythm. These results might broaden our understanding of how septum–DBS alleviates epileptic seizure and provide evidence to precisely treat clinical refractory epilepsy.

## 2 MATERIALS AND METHODS

### 2.1 Animals

*Wild‐type* mice (WT, male, RRID: IMSR_JAX:000664, 8–9 weeks old) were purchased from SLAC Laboratory Animal Center (Zhejiang, China). Choline acetyltransferase (ChAT) Cre‐recombinase mice (*ChAT‐Cre*, male, strain No. 006410, RRID: IMSR_JAX:006410, 8–9 weeks old) were used and genotyped according to the protocols provided by Jackson Laboratory. For genotyping of *ChAT‐Cre* mice, forward primer: ACC TGA TGG ACA TGT TCA GGG ATC G, reverse primer: GTT ATT CGG ATC ATC AGC TAC ACC were used. Both the positive allele PCR products were 350 bp. The program included 94°C for 3 min (1×), 94°C for 30 s, 55°C for 60 s, 72°C for 60 s (36×), and 72°C for 2 min (1×).

All the mice were housed in SPF standard conditions with a 12 h light/dark cycle (temperature 20–26°C, relative humidity 50%–60%) and free access to food and water. All behavioral experiments were carried out between 9:00 and 18:00. All procedures complied with the standards of Institutional Animal Care and were approved by the ethical committee of Zhejiang Chinese Medical University. Animal studies are reported in compliance with ARRIE guidelines.

### 2.2 Stereotaxic surgery for virus injection and electrode implantation

Mice were anesthetized with sodium pentobarbital (60 mg/kg, i.p.) and mounted in a stereotaxic apparatus (RWD Life Science Co., Ltd. Shenzhen, China). Then, the skull surface was exposed and wiped the pericranium with 3% (w/v) hydrogen peroxide. The burr holes were stereotactically made on the skull. A homemade cannula (Cat# 62004, RWD Life Science, China)‐electrode (each 0.125 mm in diameter, 0.5 mm tip distance, A‐M Systems, WA, USA) was implanted into the right dorsal hippocampus CA1 (dCA1, AP: −2.0 mm, ML: −1.3 mm, DV: −1.6 mm) for KA injection and EEG recording, and an electrode was implanted into the MS (AP: +1.1 mm, ML: 0.0 mm, DV: −4.3 mm), VDB (AP: +1.4 mm, ML: 0.0 mm, DV: −5.0 mm), or HDB (AP: +0.5 mm, ML: −0.7 mm, DV: −5.5 mm) for DBS treatment. Four screws were attached to the skull over the cortex to secure the dental cement, two of which were attached over the cerebellum to serve as the reference and ground electrodes. Mice were recovered for 1 week after surgery.

To selectively induce cholinergic neuron apoptosis in the MS or VDB, the viral injection process was performed similarly to our previous study.^32^ *rAAV‐EF1a‐flex‐taCasp3‐TEVP* (serotype: AAV2/9, viral titers: 5.62E + 12 v.g./mL, 300 nL; BrainVTA Co., Ltd, Wuhan, China) was injected into the MS or VDB subregion in *ChAT‐Cre* mice with a 1‐μL microliter syringes (Aijuyi Glass Instrument Co., Ltd. Ningbo, China) at 60 nL/min using an ultramicro pump (Micro 4, World Precision Instruments, USA). The needle was removed 5 min after the injection to prevent the virus reflux. Mice were sent back to home cage after surgery and sustained 4 weeks for maximal viral expression.

### 2.3 KA‐induced seizure model

KA‐induced seizure model was performed as our previous studies.^29^, ^33^ After 1 week of recovery, mice were administered 500 nL KA (0.5 μg/μL, dissolved in 0.9% saline, Cat# ab120100, Abcam, Cambridge, UK) through the cannula over 5 min with a 1‐μL microliter syringe. EEGs were recorded for 90 min after KA injection by the Powerlab (PowerLab 8/35, AD Instruments, Australia) and analyzed by Labchart 8.^20^ Seizure severity was scored according to the modified Racine scale^34^: (1) facial movement; (2) head nodding; (3) unilateral forelimb clonus; (4) bilateral forelimb clonus and rearing; and (5) rearing and falling. Stages 1–3 were regarded as focal seizures (FSs), and stages 4–6 were regarded as generalized seizures (GSs). Typically, mice experienced GS in tens of minutes after KA injection. The latency to stage 2 and stage 4, seizure stage, number, and duration of GS were observed and measured by researchers that blinded to the group allocation.

To investigate the effect of DBS in the different septal subregions on the KA‐induced seizure, mice received DBS treatment immediately after KA injection according to following parameters: monophasic square‐wave pulses, 0.1 mA, 0.1 ms per pulse, 5 Hz.^20^ DBS was delivered by a constant current stimulator (FE180, AD Instruments, Australia). The mice in the sham group were left in the chamber and connected to the apparatus without DBS delivery.

To calculate the power of each rhythm in EEG periods, we analyzed 30 min of hippocampal EEG before DBS modulation as the baseline and three 30‐min periods after DBS modulation as the postline under physiological conditions. The normalized power ratio of each mouse was obtained by postpower (postline) divided by prepower (baseline) to minimize individual variation. For the power of KA states, we analyzed 10 min of hippocampus EEG periods as “pre” and 90‐min periods after DBS modulation and calculated the average power ratio as “DBS.”

### 2.4 Immunohistology

Mice were deeply anesthetized with sodium pentobarbital and transcardially perfused sequentially with phosphate buffer (PBS, PH 7.4) and 4% (w/v) paraformaldehyde (PFA). After removing the brains and postfixing them in 4% PFA at 4°C overnight, we dehydrated them in 30% (w/v) sucrose for 48 h. Coronal sections of 35 μm were obtained with a sliding freezing microtome (CryoStar NX70, Thermo Fisher Scientific, CA, USA). Brain slices were floated in PBS and retrieved antigens with 0.24% sodium citrate and 0.038% citric acid in ddH_2_O. After blocking with 5% donkey serum (Cat# 36116ES10, YEASEN, Shanghai, China) in 0.3% Triton X‐100/PBS for 2 h at room temperature, the slices were incubated with the following primary antibodies overnight at 4°C: goat anti‐ChAT (1:100, Cat# AB143, RRID: AB_2079760 Millipore, MA, USA) or mouse anti‐c‐Fos (1:1000, Cat# AB208942, RRID: AB_2747772, Abcam, Cambridge, UK). The slices were rinsed with PBS three times at next day and incubated with the following secondary antibodies for 2 h at room temperature in the dark: Alexa Fluor 488 secondary antibodies (1:500, Cat# AB150105, RRID: AB_2732856, Abcam, Cambridge, UK) or Alexa Fluor 647 secondary antibodies (1:500, Cat# AB150131, RRID: AB_2732857, Abcam, Cambridge, UK). After rinsing the slices three times, we counterstained the nuclei with Fluoromount‐G™ with DAPI (Cat# 36308ES20, RRID: AB_2636877, YEASEN, Shanghai, China) and captured the images with a Leica TCS SP8 confocal laser scanning microscope (Leica Microsystems, Mannheim, Germany) and LAS X software (Version 3.7.1). For calculating the number of c‐Fos^+^ cells, we selected three consecutive coronal sections and took the average. The statistical areas of c‐Fos were a 500 * 300 μm rectangular for CA1 and CA3 and a 300 * 200 μm rectangular for DG. The c‐Fos^+^ neurons were collected and counted by the researcher blind to the group allocation.

### 2.5 Statistics

All data were presented as mean ± SEM. The number of experimental replicates (n) was indicated in each figure legend. Statistical comparisons were performed by Prism (version 9.0, GraphPad Software, CA, USA) and SPSS (version 25.0, Chicago, IL, USA) with appropriate methods indicated in figure legends. Probability (*p*) values less than 0.05 were considered statistically significant.

## 3 RESULTS

### 3.1 DBS in the MS and VDB subregions alleviates KA‐induced seizure

**FIGURE 1:**
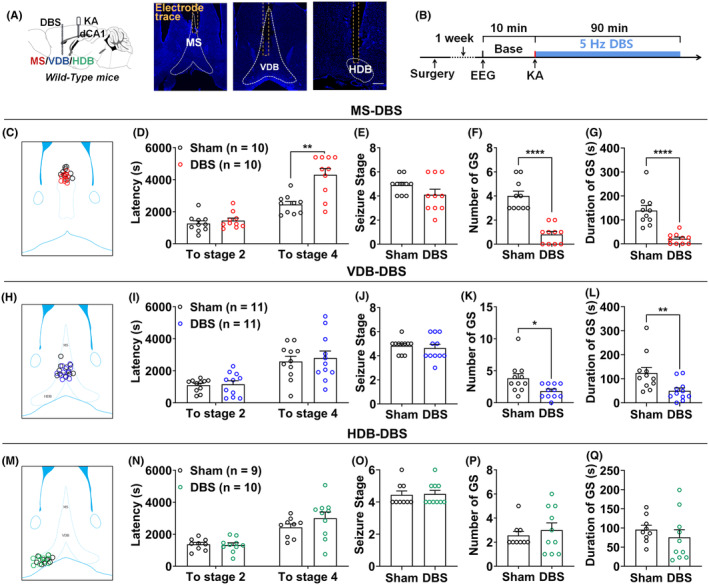
DBS in the MS and VDB subregions alleviate KA‐induced seizures. (A) Left panel, experimental diagram of electrodes implanted into the different septal subregions for DBS modulation and a homemade cannula electrode implanted into the dCA1 for KA injection and EEG recording. Right panel, representative imaging of the electrode track in the different septal subregions, scale bar: 200 μm (blue: DAPI). (B) Schematic diagram of the DBS in different septal subregions in KA‐induced seizure model. (C) All the electrode traces in the MS region (black: sham group, red: MS‐DBS group). (D–G) Effects of DBS in the MS on (D) the latency to stage 2, stage 4, (E) seizure stage, (F) number, and (G) duration of GS during the 90‐min observation period. (H) All the electrode traces in VDB (black: sham group, blue: VDB‐DBS group). (I–L) Effects of DBS in the VDB on (I) the latency to stage 2, stage 4, (J) seizure stage, (K) number, and (L) duration of GS during the 90‐min observation period. (M) All the electrode traces in HDB (black: sham group, green: HDB‐DBS group). (N–Q) Effects of DBS in the HDB on (N) the latency to stage 2, stage 4, (O) seizure stage, (P) number, and (Q) duration of GS during the 90‐min observation period. Data are presented as means ± SEM. The number of mice used in each group was indicated in the figures. *p < 0.05, **p < 0.01, and ****p < 0.0001 were compared to the sham group. N and Q were analyzed by the unpaired *t*‐test, and others were used by the Mann–Whitney test.

We first examined whether DBS in the different septal subregions showed anti‐seizure effects in KA‐induced seizure model (Figure 1A,B). Terminal positions of all electrodes for DBS have been postverified and schematically diagramed in Figure 1C (MS), H (VDB), and M (HDB), respectively. We found that DBS in the MS subregion delayed the latency to stage 4 (GS) from (2453.60 ± 185.60) s to (4303.70 ± 402.51) s (Figure 1D, *p* < 0.01, *n* = 10). Particularly, DBS in the MS decreased the number of GS from (4.00 ± 0.39) to (0.80 ± 0.25) (Figure 1F, *p* < 0.0001, *n* = 10) and reduced the total duration of GS from (138.80 ± 21.23) s to (20.90 ± 7.05) s (Figure 1G, *p* < 0.0001, *n* = 10) compared to the sham group. However, DBS in the MS did not affect the latency to stage 2 (FS) and the final seizure stage (Figure 1D,E, *p* > 0.05, *n* = 10), indicating DBS in the MS mainly delays the seizure spread and decreases the severity of GS expression. As for the DBS in the VDB subregion, it also significantly reduced the number of GS from (3.82 ± 0.72) to (1.82 ± 0.33) (Figure 1K, *p* < 0.05, *n* = 11) and the duration of GS from (123.82 ± 23.50) s to (49.64 ± 12.38) s (Figure 1L, *p* < 0.01, *n* = 11), but limitedly affected the latency to stage 2, stage 4, and the final seizure stage (Figure 1I–J, *p* > 0.05, *n* = 11), suggesting DBS in the VDB only reduced severity of GS expression, while for DBS in the HDB subregion, there was no significant difference (Figure 1N–Q, *p* > 0.05).

### 3.2 DBS in the MS and VDB subregions decrease neural excitability in the hippocampus in KA‐induced seizure model

**FIGURE 2:**
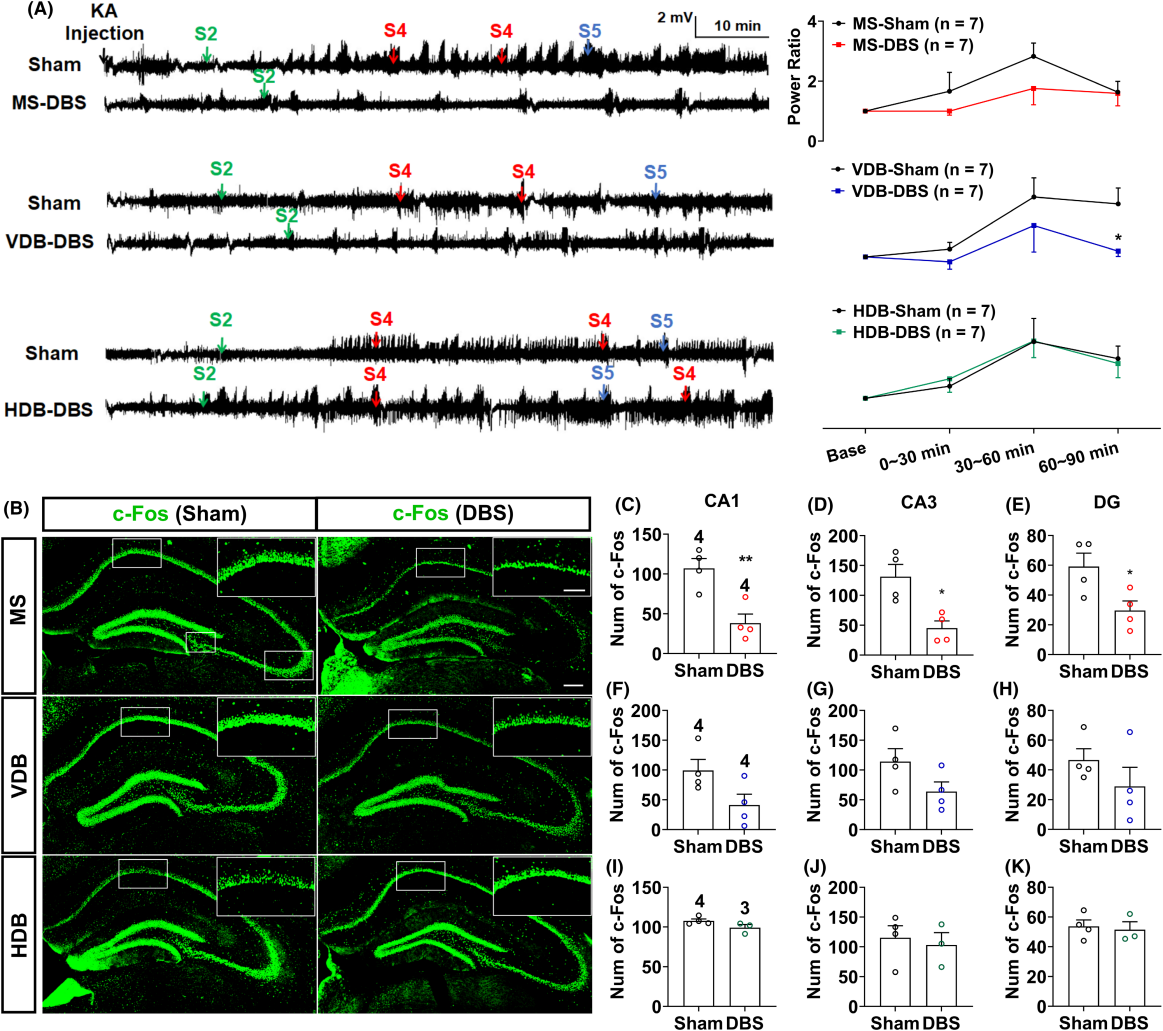
DBS in the MS and VDB subregions decreases neural excitability in the hippocampus in the KA‐induced seizure model. (A) Representative EEGs and corresponding quantification of EEG power spectra from different groups in the KA‐induced seizure model. (B) Representative imaging of c‐Fos (green) staining of contralateral hippocampus in the different groups, scale bar: 200 μm. The statistical areas of c‐Fos were marked in the enlarged imaging, a 500 * 300 μm rectangular for CA1, scale bar: 400 μm. (C–E) Quantification of MS‐DBS on the numbers of c‐Fos^+^ neurons in (C) CA1, (D) CA3, and (E) DG. (F–H) Quantification of VDB‐DBS on the numbers of c‐Fos^+^ neurons in (F) CA1, (G) CA3, and (H) DG. (I–K) Quantification of HDB‐DBS on the numbers of c‐Fos^+^ neurons in (I) CA1, (J) CA3, and (K) DG. Data are presented as means ± SEM. The number of mice used in each group was indicated in the figures. *p < 0.05 and **p < 0.01 were compared to the sham group. (A) was analyzed by the two‐way ANOVA followed by Tukey's multiple comparison test and C–K were analyzed by the unpaired *t*‐test.

It was previously reported that the septal‐hippocampal circuit ameliorated TLE by preferentially decreasing the excitability of hippocampal somatostatin‐positive neurons.^29^ To investigate whether the DBS in the MS and VDB subregions modulated the excitability of the hippocampus, the KA‐injection site, we analyzed EEGs recording in the dorsal hippocampal CA1 (dCA1). The results showed that DBS in the MS had a tendency to decrease relative power ratio from (1.65 ± 0.58) to (0.92 ± 0.13) (Figure 2A, *p* > 0.05, *n* = 7) after 30‐min DBS and from (2.61 ± 0.41) to (1.70 ± 0.50) (Figure 2A, *p* > 0.05, *n* = 7) after 60‐min DBS, while DBS in the VDB reduced relative power ratio from (1.17 ± 0.21) to (0.81 ± 0.23) (Figure 2A, *p* > 0.05, *n* = 7) after 30‐min DBS, from (2.82 ± 0.59) to (2.06 ± 0.82) (Figure 2A, *p* > 0.05, *n* = 7) after 60‐min DBS, and from (2.58 ± 0.49) to (1.1 ± 0.16) (Figure 2A, *p* < 0.05, *n* = 7) after 90‐min DBS. DBS in the HDB showed similar total power with the sham group (*n* = 7). Discrepant effects of DBS in the septal subregions could also be showcased in the corresponding EEGs and power spectra.

In addition, we applied immunohistochemical staining of c‐Fos, an expression product of the immediate early gene *fos* that is easily activated by epileptic seizures.^35^ The results revealed that DBS in the MS decreased the number of c‐Fos^+^ cells in the CA1 from (107.08 ± 12.19) to (38.25 ± 11.35) (Figure 2C, *F* (3) = 1.04, *p* < 0.01, *n* = 4), CA3 from (131.23 ± 20.56) to (45.33 ± 11.98) (Figure 2D, *F* (3) = 1.82, *p* < 0.05, *n* = 4), and DG from (59.10 ± 9.01) to (29.63 ± 6.31) (Figure 2E, *F* (3) = 2.9, *p* < 0.05, *n* = 4) after KA‐induced seizure compared with those without DBS. DBS in the VDB slightly decreased the number of c‐Fos^+^ cells in the CA1 from (99.33 ± 18.62) to (41.22 ± 18.26), CA3 from (114.17 ± 21.83) to (63.78 ± 16.18), and DG from (46.73 ± 7.53) to (28.93 ± 12.82) (Figure 2F–H, *p* > 0.05, *n* = 4) after KA‐induced seizure compared with the sham group. In contrast, HDB‐DBS did not change the number of c‐Fos^+^ cells within the hippocampus (Figure 2I–K, *p* > 0.05) compared with the sham.

### 3.3 DBS in the MS and VDB regulates the hippocampal rhythm

**FIGURE 3:**
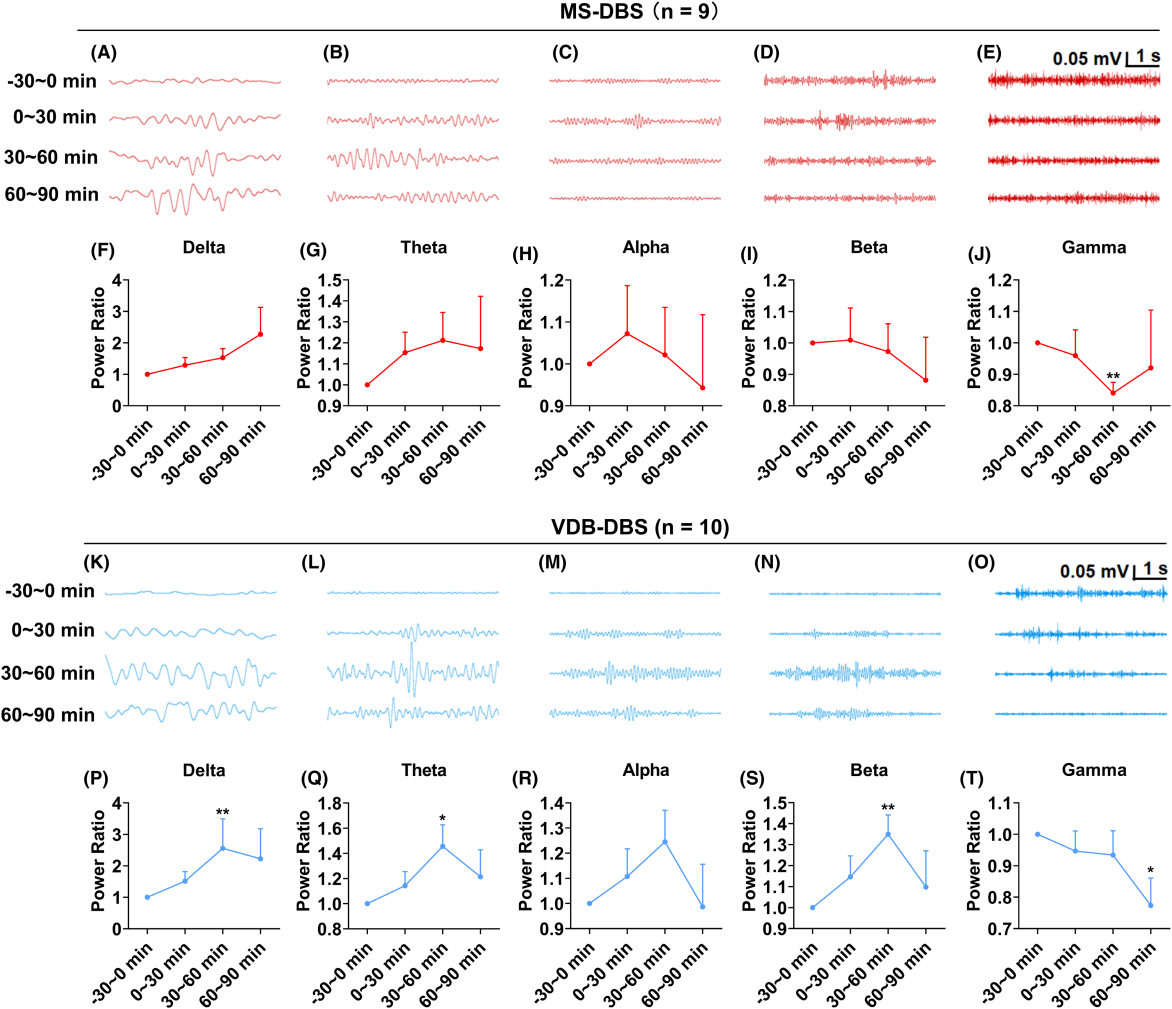
DBS in the MS and VDB subregions modulate the hippocampal basal EEG rhythm. (A–E) Representative hippocampal EEGs with (A) delta, (B) theta, (C) alpha, (D) beta, and (E) gamma rhythm bands at different time points. (F–J) Quantification of EEG power spectra of MS‐DBS on hippocampal (F) delta, (G) theta, (H) alpha, (I) beta, and (J) gamma rhythms. (K–O) Representative hippocampal EEGs with (K) delta, (L) theta, (M) alpha, (N) beta, and (O) gamma rhythms at different times. (P–T) Quantification of EEG power spectra of VDB‐DBS on hippocampal (P) delta, (Q) theta, (R) alpha, (S) beta, and (T) gamma rhythms. Data are presented as means ± SEM. The number of mice used in each group was indicated in the figures. *p < 0.05 and **p < 0.01 compared to the baseline. F–J and P–T were analyzed by the Wilcoxon test.

Next, we tested how DBS in the MS and VDB subregions modulates neural excitability by further analyzing the hippocampal EEG rhythm in physiological state. Indicated by representative hippocampal EEGs and rhythms in Figure 3A–E, DBS in the MS had a tendency to increase the power ratio of delta and theta oscillation (Figure 3F,G) and lower the alpha, beta, and gamma oscillations (Figure 3H–J). Notably, the power ratio of gamma oscillation was significantly decreased (Figure 3J, *p* < 0.001, *n* = 9) after receiving DBS compared to the baseline (−30 ~0 min). As for the VDB, DBS showed similar changes in hippocampal rhythm (Figure 3K–O), that is, improving the power ratio of delta oscillation (Figure 3P, *p* < 0.01, *n* = 10), theta oscillation (Figure 3Q, *p* < 0.05, *n* = 10) and decreasing gamma oscillation (Figure 3T, *p* < 0.05, *n* = 10). Slightly differently, DBS in the VDB augmented beta oscillation, in contrast to that in the MS.

### 3.4 Septal cholinergic neurons mediate the anti‐seizure and EEG‐modulating effects of DBS in the MS and VDB

**FIGURE 4:**
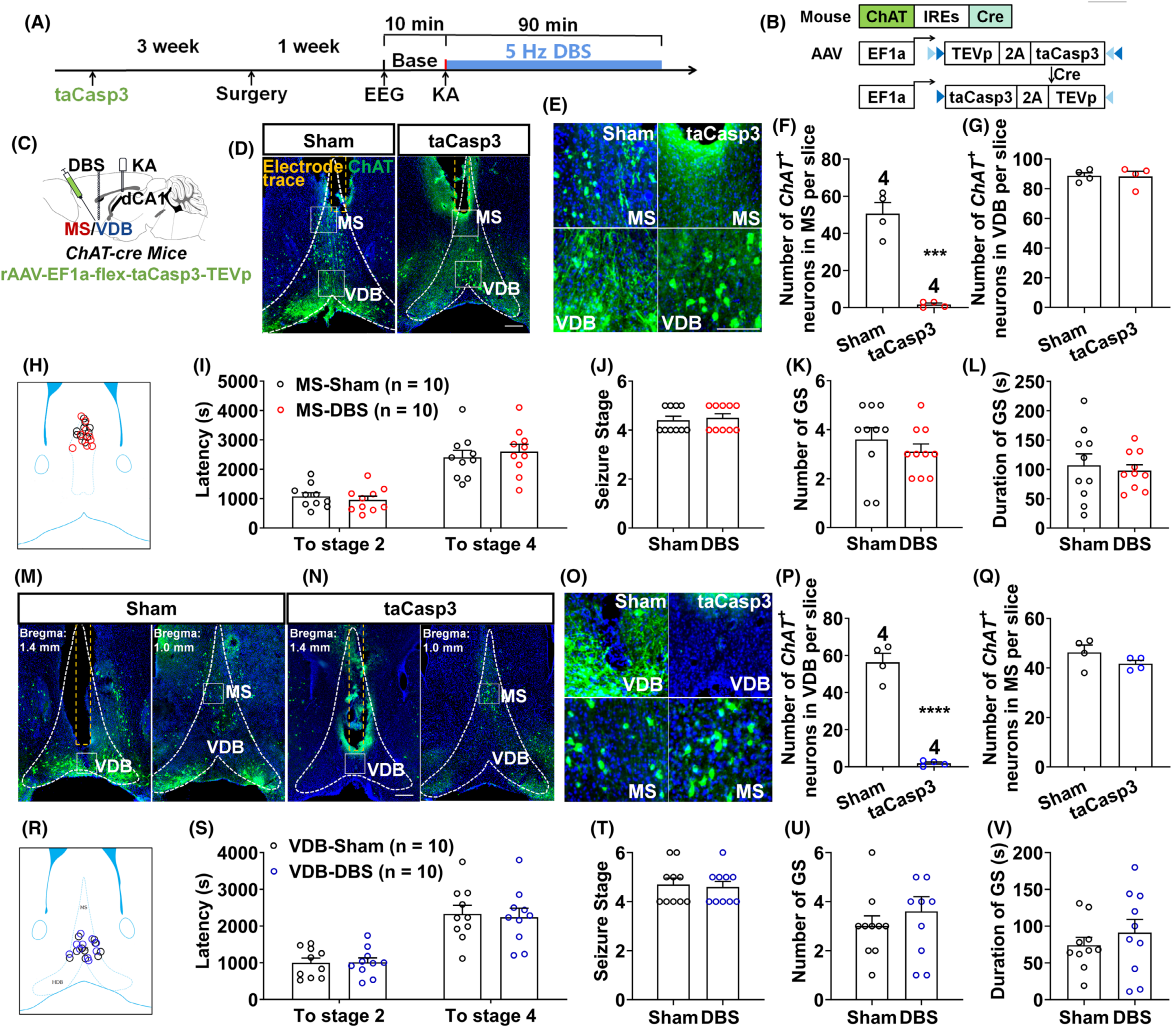
Ablation of cholinergic neurons blocks the anti‐seizure effect of DBS in the MS and VDB subregions. (A) Schematic diagram of the virus injection (cholinergic neuron apoptosis) and DBS in the MS or VDB subregions in the KA‐induced seizure model. (B) *rAAV‐EF1a‐flex‐taCasp3* was injected in the MS or VDB subregions of *ChAT‐Cre* mice to induce MS or VDB cholinergic neuron apoptosis, respectively. (C) Scheme of experiment for virus injection, DBS treatment, and KA injection. (D, E) Representative image of the immunostaining of ChAT^+^ (green) neuron in the (D) Sham and taCasp3 group after the ablation of cholinergic neurons in MS subregions, and (E) the enlarged views of the selected regions in the (D). Scale bar, 200 μm and 30 μm (blue: DAPI). (F, G) Corresponding statistics of the number of cholinergic neurons in (F) MS and (G) VDB. (H) All the electrode traces in the MS (black: Sham group, blue: MS‐DBS group). (I–L) Effects of DBS in the MS on (I) the latency to stage 2, stage 4, (J) seizure stage, (K) number, and (L) duration of GS during the 90‐min observation period. (M–O) Representative images of the immunostaining of ChAT^+^ (green) neuron in the (M) Sham and (N) taCasp3 group at the VDB (left) and MS (right, shown in the Bregma: +1.0 mm as the level of the Bregma: +1.4 mm was damaged by the electrode trace of the VDB‐DBS) after the ablation of cholinergic neurons in VDB subregions, and (O) the enlarged views of the selected regions in the (M, N). Scale bar, 200 μm and 30 μm (blue: DAPI). (P, Q) Corresponding statistics of the number of cholinergic neurons in (P) VDB and (Q) MS. (R) All the electrode traces in the VDB (black: sham group, blue: VDB‐DBS group). (S–V) Effects of DBS in VDB on (S) the latency to stage 2, stage 4, (T) seizure stage, (U) number, and (V) duration of GS during the 90‐min observation period. Data are presented as means ± SEM. The number of mice used in each group was indicated in the figures. ***p < 0.001 and ****p < 0.0001 were compared to the sham group. F, G, P, Q, I, L, S, and V were analyzed by the unpaired *t*‐test, and J, K, T, and U were used by the Mann–Whitney test.

Previous studies have reported that induction of theta frequency oscillation, which is internally decreased in epileptic conditions, could contribute to seizure alleviation.^36^, ^37^ With regard to the hippocampal theta oscillation, it is generally acknowledged to be primarily dependent on septal cholinergic inputs.^38^ Therefore, to investigate whether cholinergic neurons are involved in the anti‐seizure effects of DBS in the MS or VDB, we injected *rAAV‐EF1a‐flex‐taCasp3* into the MS or VDB of *ChAT‐Cre* mice to selectively induce cholinergic neuron apoptosis (Figure 4A–C). The immunochemistry results (Figure 4D,E) showed a 92.55% loss of cholinergic neurons in the MS from (50.61 ± 6.02) to (1.64 ± 0.79) (Figure 4F, *p* < 0.001, *n* = 4), verifying successful killing without affecting the VDB or other brain regions (Figure 4G, *p* > 0.05, *n* = 4). As before, all the terminal positions of electrodes had been checked, and the schematic diagram of electrode positions is shown in Figure 4H. In the absence of cholinergic neurons, we found that DBS in the MS did not affect the latency to seizure stages 2 and 4, the final seizure stage, number, and duration of GS (Figure 4I–L, *p* > 0.05, *n* = 10). Similarly, for the VDB cholinergic neuron apoptosis, immunochemistry results verified that the cholinergic neuron loss in the VDB (Figure 4M–O) from (56.29 ± 4.87) to (1.86 ± 0.72) (Figure 4P, *p* < 0.01, *n* = 4), while the MS was not interfered (Figure 4Q, *p* > 0.05, *n* = 4). Terminal positions of all electrodes in the VDB were shown in Figure 4R. DBS in the VDB also did not affect the severity of seizure (Figure 4S–V, *p* > 0.05, *n* = 10) after killing the VDB cholinergic neurons.

**FIGURE 5:**
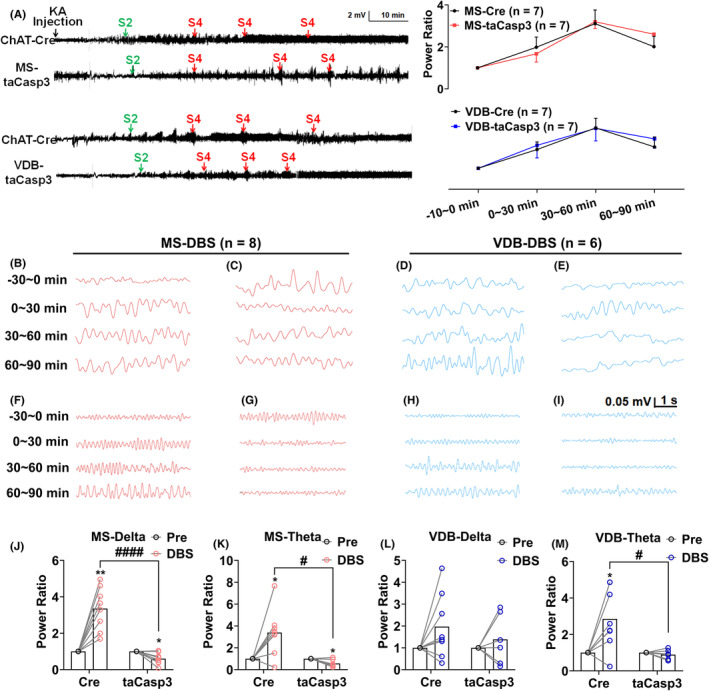
Ablation of cholinergic neurons blocks EEG‐modulating effects of DBS in the MS and VDB subregions. (A) Representative EEGs and corresponding quantification of EEG power spectra from different groups in KA‐induced seizure model after ablation of cholinergic neurons. Representative EEGs of hippocampal (B–E) delta and (F–I) theta rhythm and (J–M) corresponding power ratio after ablation of cholinergic neuron in the MS (red) and VDB (blue). Data are presented as means ± SEM. *p < 0.05 and **p < 0.01 compared to the Pre. #*p* < 0.05 and ####*p* < 0.0001 compared to the Cre group. J–M were analyzed by the Wilcoxon test when compared with the Pre, J, L, and M were analyzed by the unpaired *t*‐test and K was analyzed by the Mann–Whitney test when compared with the Cre group.

Finally, to verify whether the effect of DBS on the low‐frequency rhythms of the hippocampus changed after cholinergic neuron apoptosis in the MS or VDB, we analyzed the hippocampal EEG rhythm in the KA‐induced seizure model (Figure 5A). As shown in representative EEGs in Figure 5B,C,F,G. Enhanced the power ratio of delta oscillation (Figure 5J, *p* < 0.01, *n* = 8) and theta oscillation (Figure 5K, *p* < 0.05, *n* = 8) by MS‐DBS was significantly reduced after killing the cholinergic neurons (Figure 5J,K, *p* < 0.05, *n* = 8) in the MS, suggesting these oscillations are dependent on cholinergic neurons. Similarly, we found that increased power ratio of delta and theta oscillations by VDB‐DBS was reversed after cholinergic neuron loss in the VDB (Figure 5D,E,H,I,L,M, *n* = 6).

## 4 DISCUSSION

Although the anti‐seizure effect of DBS in the septal region has been put forward by previous studies, the underlying mechanism remains somewhat controversial.^18^, ^19^, ^20^ The present study further investigated the precise targets of septal DBS and potential mechanisms. Here, we first found that 5 Hz DBS in the MS or VDB, rather than the HDB ameliorates KA‐induced seizure, which extends the previous finding that septum‐DBS has an anti‐seizure effect.^39^ Notably, we found that DBS in the MS delays the latency to the GS and reduces its severity, while DBS in the VDB only shortens the number and duration of GS. DBS in both regions did not affect the latency to stage 2, indicating that DBS in the MS may be involved in seizure generalization rather than early seizure development. Other research groups and we have demonstrated the anti‐epileptic effect of septal DBS in several rodent epilepsy model, including the pilocarpine‐induced seizure model, hippocampal kindling model, and the KA‐induced seizure model, which suggested that the septum could be a promising brain region for clinical translation.^18^, ^20^, ^40^ Additionally, recent research also shown the prevention of seizures generalization after DBS in the MS, which further supporting our results.^19^ However, the optimal subtargets for septal DBS were unclear at that time. On this basis, we designed the current study to identify the key subregion for DBS treatment. Our present data strongly indicate that DBS in the MS and VDB rather than HDB is anti‐seizure. Clinically, high‐frequency stimulation (HFS), which leads to the background localized potential desynchronization and decreases spike activity in the onset area, is widely used to treat patients with refractory epilepsy.^41^ However, prolonged HFS causes neuronal damage, increases extracellular potassium aggregation, and interrupts normal function.^42^, ^43^ Thus, a variety of experiments focused on low‐frequency stimulation (LFS). Compared to HFS, LFS not only decreased the frequency of spontaneous recurrent seizures, but could prevent excessive neuronal damage and improve cognitive impairment.^44^ Combined with our recent study,^20^ we suggested that LFS in the septum, especially the MS and VDB subregions, drives the best effect in TLE. This may be of great therapeutic significance for the clinical treatment of epilepsy with septal DBS at specific subregions. Furthermore, the side effects are usually mentioned in various researches on DBS. One common side effect of DBS is cerebral hemorrhage or infection induced by electrode implantation.^45^ As the septum locates in the midline, a simpler and safer electrode insertion procedure would reduce the aforementioned risk.^39^ Furthermore, disadvantages come from the tight connections between the MS and the limbic system. Whether septal DBS may cause cognitive side effects due to the close connection with hippocampus is still not clear. However, our previous study has showed the cognitive improvement of MS‐DBS.^20^ Whether the complex connection between the septum and other nuclei of the limbic system could also lead to previsible side effects needs further exploration.

As the main output region of the septum, the hippocampus is also the most commonly seen focus in TLE.^15^ Our results demonstrated that DBS in the MS or VDB subregions directly reversed the aberrant neuronal activation in the hippocampus induced by KA. In addition, we also found that DBS in the MS or VDB modulated the hippocampal rhythms during both the physiological and epileptic conditions. It is worth our attention that DBS in the MS or VDB especially enhances the hippocampal theta rhythms, in line with our previous findings, confirming that entrainment of theta rhythms effectively alleviates seizures in TLE.^20^ In fact, reduction of theta power in the MS‐hippocampal circuit, which was accompanied by a mild decrease in the theta frequency in the hippocampus of awake epileptic animals, was found to be epileptic in both rodent pilocarpine and KA models.^46^, ^47^ Additionally, our results indicated that DBS in the MS and VDB significantly decreased the power ratio of gamma oscillation, while DBS in the VDB augmented beta oscillation. Previous research has also reported that seizure activities could cause the increase of high‐amplitude hippocampal gamma waves, which may be a key index of the aggravated seizure severity, whereas the beta rhythms could have an anti‐epileptic effect.^48^, ^49^ Accordingly, we think the decrease of gamma oscillation and increase of beta oscillation after DBS in the MS and VDB could be both the potential mechanism to attenuate KA‐induced seizure. We should also note that an increase of delta band was found in VDB‐DBS, suggesting a wide range of spectrum was influenced by DBS. Specifically, as the hippocampal theta oscillation is widely recognized to be mediated by septal cholinergic input,^50^ we next verified whether cholinergic neurons in the MS or VDB determined the anti‐seizure effect of DBS. To achieve this, we repeated the DBS treatment in the MS or VDB after the ablation of local cholinergic neurons in the corresponding subregion in KA‐induced seizure mice, the results showed that anti‐seizure effect and the enhancement of hippocampal theta power mediated by DBS were reversed, indicating the importance of septal cholinergic signaling in driving theta rhythm and thus seizure controlling. Moreover, the anti‐inflammatory effects of DBS in epilepsy remission were also reported in recent studies. The reduction of the mRNA expression of IL‐1β, IL‐6, and TNF‐α was found after DBS in rat KA model, which breaking the vicious cycle of blood–brain barrier disruption and albumin extravasation, thus further alleviating the seizure.^51^ Additionally, the biological activity of astrocytes was also found to be reduced in rat PTZ model, and it may due to the inhibitory effects of DBS on pre‐inflammatory factors.^52^ Whether DBS in the MS and VDB also recruited neuroinflammatory pathway worth further study.

Although sharing quite a lot similar characteristics, more sophisticated heterogeneity in septal cholinergic neurons were reported by recent advances. An electrophysiological study divides them into early and late‐firing neurons and puts forward that these two distinct cholinergic populations may be involved in different physiological functions.^53^ Indeed, a recent study has reported that cholinergic neurons in the MS could be divided into two subsets according to the presence or absence of *D28K* gene. These two subpopulations of cholinergic neurons have different morphological structures, electrophysiological properties, and mutually exclusive marker genes. Their neural connections with the hippocampus and functions were also biased,^54^ indicating research insight into more details in septal cholinergic neurons. Our present results strongly supported this idea, suggesting that the anti‐seizure function of septal DBS came from cholinergic neurons in the MS and VDB, rather than the HDB mediated theta rhythms. The precise difference in genetic constitution and circuit organization of cholinergic neurons in these septal subregions worth further exploration.

In conclusion, our results demonstrated that DBS in the MS and VDB rather than HDB attenuates KA‐induced seizure by enhancing the hippocampal theta rhythms through the cholinergic neurons. These results may shed light on the importance of precise regulation of DBS therapy in treating epileptic seizures.

## AUTHOR CONTRIBUTIONS

YPR, JJF, and FF designed the research. QYZ, YuW, FW, DXJ, and YJS conducted the most of the experiments. QYZ and FW performed the data analysis. YiW, YuW, LY, and MDZ provided constructive advice and contributed to the discussion. YiW, FF, and YuW revised the manuscript and supervised all aspects of the work.

## CONFLICT OF INTEREST STATEMENT

None of the authors has any conflict of interest to disclose. We confirm that we have read the Journal's position on issues involved in ethical publication and affirm that this report is consistent with those guidelines.

## Data Availability

The authors declare that all data used in this study are available from the corresponding author by reasonable request.
